# A Locomotor Deficit Induced by Sublethal Doses of Pyrethroid and Neonicotinoid Insecticides in the Honeybee *Apis mellifera*

**DOI:** 10.1371/journal.pone.0144879

**Published:** 2015-12-14

**Authors:** Mercédès Charreton, Axel Decourtye, Mickaël Henry, Guy Rodet, Jean-Christophe Sandoz, Pierre Charnet, Claude Collet

**Affiliations:** 1 INRA, UR 406 Abeilles et Environnement, 84914, Avignon, France; 2 UMT, Protection des Abeilles dans l’Environnement, 84914, Avignon, France; 3 ITSAP-Institut de l’abeille, 84914, Avignon, France; 4 ACTA, 84914, Avignon, France; 5 CNRS, Univ Paris-Sud, IRD, UMR 9191 Evolution, Genomes, Behavior and Ecology, 91198, Gif-sur-Yvette, France; 6 CNRS, UMR 5237, Université Montpellier 2, Centre de Recherche de Biochimie Macromoléculaire, 34293, Montpellier, France; University of California, San Diego, UNITED STATES

## Abstract

The toxicity of pesticides used in agriculture towards non-targeted organisms and especially pollinators has recently drawn the attention from a broad scientific community. Increased honeybee mortality observed worldwide certainly contributes to this interest. The potential role of several neurotoxic insecticides in triggering or potentiating honeybee mortality was considered, in particular phenylpyrazoles and neonicotinoids, given that they are widely used and highly toxic for insects. Along with their ability to kill insects at lethal doses, they can compromise survival at sublethal doses by producing subtle deleterious effects. In this study, we compared the bee’s locomotor ability, which is crucial for many tasks within the hive (e.g. cleaning brood cells, feeding larvae…), before and after an acute sublethal exposure to one insecticide belonging to the two insecticide classes, fipronil and thiamethoxam. Additionally, we examined the locomotor ability after exposure to pyrethroids, an older chemical insecticide class still widely used and known to be highly toxic to bees as well. Our study focused on young bees (day 1 after emergence) since (i) few studies are available on locomotion at this stage and (ii) in recent years, pesticides have been reported to accumulate in different hive matrices, where young bees undergo their early development. At sublethal doses (SLD_48h_, i.e. causing no mortality at 48h), three pyrethroids, namely cypermethrin (2.5 ng/bee), tetramethrin (70 ng/bee), tau-fluvalinate (33 ng/bee) and the neonicotinoid thiamethoxam (3.8 ng/bee) caused a locomotor deficit in honeybees. While the SLD_48h_ of fipronil (a phenylpyrazole, 0.5 ng/bee) had no measurable effect on locomotion, we observed high mortality several days after exposure, an effect that was not observed with the other insecticides. Although locomotor deficits observed in the sublethal range of pyrethroids and thiamethoxam would suggest deleterious effects in the field, the case of fipronil demonstrates that toxicity evaluation requires information on multiple endpoints (e.g. long term survival) to fully address pesticides risks for honeybees. Pyrethroid-induced locomotor deficits are discussed in light of recent advances regarding their mode of action on honeybee ion channels and current structure-function studies.

## Introduction

Pollinators play a crucial role in maintaining vegetal biodiversity but also participate in improving agricultural production. Therefore, a number of managed honeybee colonies are periodically moved in the vicinity of agricultural fields, not only to increase honey production but to improve crop pollination as well. As a consequence, insecticide exposure of honeybee colonies does not only occur when individuals are foraging, but also happens directly in the hive, as demonstrated by measurements of in-hive pesticide residues [1, 2]. In the last few decades, an increase in honeybee colony mortalities has been reported around the world and focused the attention of a broad scientific community on the potential consequences of pesticide misuse on pollinator survival [3–5]. These studies have been especially focused on two families of insecticides, neonicotinoids and phenylpyrazoles, owing to their use as systemic insecticides in seed treatment [6]. Recently, the sublethal toxicity of neonicotinoids towards honeybees has been demonstrated in real-world environments and led the European Union to restrict the use of three members of this class for two years [7–9]. Similarly, fipronil, a phenylpyrazole highly toxic to bees even at sublethal levels (by impairing memory and synergistically enhancing sensitivity to the pathogen Nosema [10, 11]) has also been banned as an agrochemical product in France and more recently in other countries of the European Union, although it is still widely used elsewhere, like the neonicotinoids [12]. It is worth mentioning that these temporary restrictions apply for seed coating only, whereas other agrochemical formulations are still authorized (Official Journal of the European Union OJ L219/22–15.8.2013 and OJ L139/12–25.5.2013). Besides neonicotinoids and phenylpyrazoles, pyrethroid insecticides constitute a large insecticide family produced through chemical synthesis, with a limited number of compounds (e.g. deltamethrin, cypermethrin, λ-cyalothrin, permethrin) accounting for the majority of sales [13]. The restrictions imposed on neonicotinoids and phenylpyrazoles may lead to an increase in pyrethroid use. Many pyrethroids are also highly toxic towards honeybees [14], and very few studies have compared the sublethal toxicities of pyrethroids, neonicotinoids and phenylpyrazoles in honeybees [15, 16]. These insecticides all target ion channels involved in the function of a variety of tissues (including the nervous and the muscular systems), and it is known that their primary mode of action is to interfere with the normal function of voltage-gated sodium channels (for pyrethroids), nicotinic acetylcholine receptors (for neonicotinoids) and glutamate and GABA receptors (for phenylpyrazoles).

The effects of sublethal doses of insecticides on the neuromuscular system of honeybees are not easy to analyze. Methods for evaluating the ability of bees to fly back to the hive after exposure to a sublethal dose of insecticide (the ‘homing flight assay’) have been recently developed [7, 17, 18]. Besides the importance of flight for bees, efficient ambulation (walking) inside the hive is required for many tasks, including cell construction and cleaning, larval feeding and social interactions in general [19]. Muscle contraction, allowing physical movements, also produces heat [20] and thus participates in maintaining proper temperature levels around the brood. In feral colonies and in managed hives, the combs, built vertically, add an additional physical challenge by requiring vertical displacements. Experimentally, evaluation of locomotor abilities inside the hive is challenging and requires special observation hives with glass sides. Locomotion assays in laboratory conditions (open-field arena) are easier to set-up and produce a simple standardized and reproducible test to evaluate the effect of sublethal doses of insecticides [21]. Sublethal doses of neonicotinoids can (acetamiprid 0.1 μg/bee) or cannot (thiamethoxam 1 ng/bee) modify walking locomotion [22]. Whereas chronic exposure to thiamethoxam or imidacloprid sublethal concentrations (24h, 10 nM) did not modify the walking behavior, the righting reflex was affected [23]. Imidacloprid sublethal doses reduce waggle dancing 24 h after ingestion [24]. Low doses of phenylpyrazole (fipronil 1 ng/bee) modify behavior (impaired olfactory learning and decreased sucrose sensitivity) without any effect on locomotion [10], while locomotion is affected by sublethal doses of one of the least toxic pyrethroids (tau-fluvalinate at a doses causing 10% mortality, LD10 [25]). Although these studies suggest that the sublethal effects of most insecticides are molecule-specific and cannot simply be extrapolated directly from the LD_50_ values, none of these molecules have been tested together in parallel tests in the same experimental conditions. Owing to the prevalence of pesticides in the vicinity of hives but also inside the hive’s matrices, locomotor deficits may affect multiple developmental stages of bees, from foragers (after contact with treated plants) to larvae and recently emerged, young bees (through contact with contaminated matrices). However, the current testing protocols only impose toxicological tests on either young larvae or unselected adults (samples shall be collected without regard to the age of the bees). While the exploration of specific toxicological effects on newly emerged bees are not yet required, this early stage of imaginal development may show a different sensitivity compared to older adults [26, 27].

The aim of this study was therefore to compare the deleterious effects of sublethal doses of several pyrethroids, one neonicotinoid and one phenylpyrazole on the locomotion of honeybees in their first day of adult life. This study shows for the first time that an acute contact exposure to sublethal doses of pyrethroid or neonicotinoid insecticides induces serious locomotor deficits in young bees that can be quantified several hours after exposure.

## Materials and Methods

### Honeybees

Newly emerged bees (*Apis mellifera*) were obtained during the spring season from two hives (with sister queens) maintained in the experimental apiary of the *Abeilles & Environnement* Research Department on the Avignon INRA PACA campus. Colonies received a treatment against *Varroa* in October (Apivar^TM^, active ingredient amitraze) and were healthy, without any obvious symptoms of disease. Thiamethoxam experiments were performed on colony 1 (summer 2013), all other experiments were performed on colony 2 (summer 2014). To collect bees, frames of developing brood were gently brushed to get rid of adult bees and placed into an incubator (30°C, high humidity) overnight in order to harvest newly emerged bees the next morning (upon emergence, bees were fed on food stored in combs).

### Exposure to insecticides

**Fig 1:**
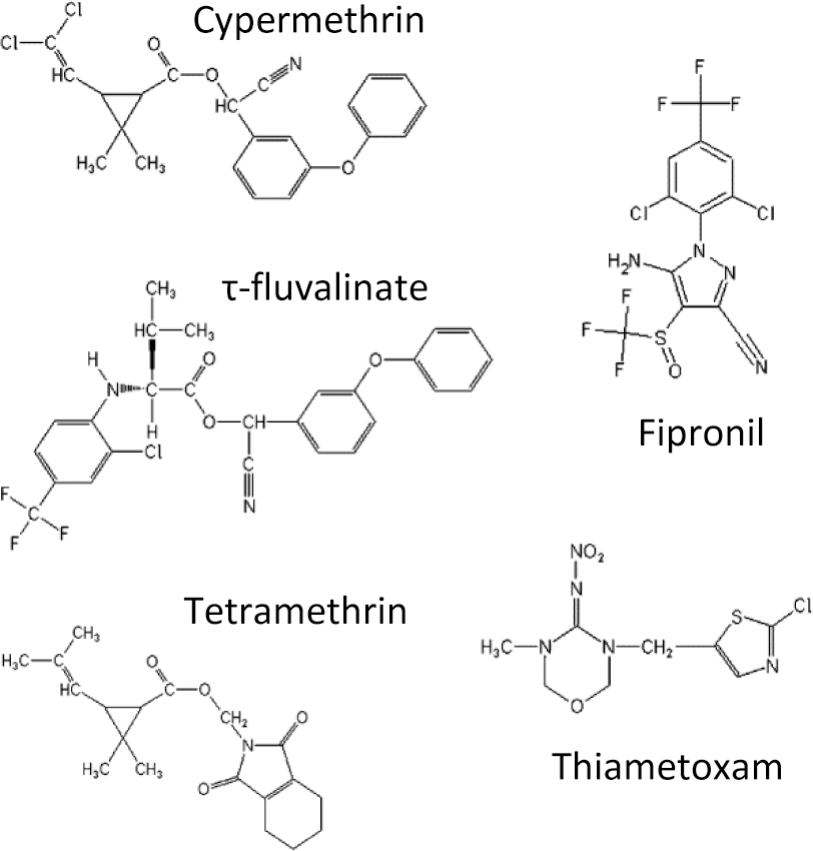
Synthetic insecticides from three classes. Chemical structure of 3 pyrethroids (cypermethrin, tau-fluvalinate, tetramethrin), a phenylpyrazole (fipronil) and a neonicotinoid (thiamethoxam).

Technical-grade insecticides (the active ingredients) were purchased from EhrenStorfer GmbH (cypermethrin, tetramethrin, tau-fluvalinate, fipronil and thiamethoxam 96, 98, 94, 97 and 98% pure, respectively). Molecules (whose molecular structure are given in Fig 1) were dissolved in acetone and final concentrations were obtained by successive dilutions in amber glass vials thoroughly vortexed at each step. Exposure to insecticides was performed between 9 and 10 am. Honeybees were anaesthetized with CO_2_ (batches of bees were exposed to a controlled volume of CO_2_ (final concentration 50%) for 30 seconds in an anesthesia induction chamber). They were placed on ice while 1 μl solution was applied to the dorsal part of the thorax with a Hamilton syringe mounted in a repeating dispenser. Full acetone evaporation was allowed and bees were placed in standard plastic cages (10.5 cm x 7.5 cm x 11.5 cm, modified from [28]) and provided with water and sugar paste (Apifonda, Ickowicz–sucrose 85%, glucose 5%, fructose 3%, water) in a ventilated incubator (29°C, 40% humidity, dark). Mortality tests were performed for all tested insecticides prior to the locomotion assay in order to determine each insecticide’s sublethal dose (SLD). A minimum of two replicates of 30 bees was used at each dose (S1 Table), which is twice the number of bees required in registration tests [26, 27]. The sublethal range was defined as doses producing a mortality level not statistically different from the control 48 hours after exposure (S1 Table). Moreover, for each insecticide, a dose two fold higher than the SLD_48h_ caused a mortality significantly higher than the control 48 hours after exposure (S1 Table). The sublethal range for each insecticide was validated on a minimum of four replicates of 30 bees (up to eight, S1 Table). Selected sublethal doses (SLD_48h_) were 2.5, 33 and 70 ng/bee for the pyrethroids cypermethrin, tau-fluvalinate and tetramethrin respectively. Selected SLD_48h_ were 0.5 and 3.8 ng/bee for fipronil and thiamethoxam, respectively. In control modalities (acetone only), mortality at 48 h did not exceed 2.5% (see Results). Control mortality was measured from a minimum of six replicates of 30 bees (up to eight). A long-term survival test (up to 5 days after exposure) was performed as well, to quantify mortality over durations longer than 48 h. If any, dead bees were removed daily from cages. Mortality at 48 h and long-term survival (at 120 h) were assessed on different bees from those assayed for locomotion.

### Video tracking analysis

Locomotor activity was monitored for 3 minutes using a webcam controlled with VirtualDub (GNU free software, acquisition frequency 1 Hz, http://sourceforge.net/projects/virtualdub/files/). The arena set up allows video tracking one bee at a time, every 5 minutes (3 minutes of effective video tracking and 2 additional minutes to transfer the bee from its cage to the arena and to allow for short time acclimation, and to transfer it back to a cage at the end of the tracking). Video tracking was performed between 2 and 6 pm and bees were allowed to recover from anesthesia during several hours in a ventilated incubator (29°C, 40% humidity, dark). For a single insecticide, control and insecticide-exposed bees were alternatively assayed and the total series duration was thus >200 minutes. Three minutes of video tracking were sufficient to characterize a distance covered at a nearly steady-state speed. The three minute duration also prevented speed adaptation (S3 Fig) that has been shown to arise quickly in some arena systems (e.g. a significant speed decrease ~10 minutes after placing the bee in the arena [29]). The vertical observation arena, inspired from existing arenas [10], measured 30 x 30 x 4 cm (height: width: depth), was illuminated from above and placed in a dark chamber to avoid any variation due to daylight. The light source consisted of two parallel flicker free LED ramps (length 10 inches, 9 LED each), for a total of 0.72 W, 70 lumens of cold light (StarLED sticks, Starlicht, Germany). Experiments were done at room temperature (22–24°C). For each insecticide, bees were taken alternatively from control and exposed groups (random selection in each case) and introduced into the arena through a hole at the bottom with entomological forceps. Videos were semi-automatically analyzed using Image J (open source, Rasband WS, National Institutes of Health, Bethesda, http://imagej.nih.gov/ij/) with available filters and plugins in order to obtain a series of x,y coordinates for each bee. Individual paths were analyzed with Excel and Origin softwares (OriginLab) and the total distance covered by insecticide-exposed bees was expressed relative to the respective mean value obtained in control bees for each pesticide.

### Statistics

Distances are expressed as mean ± S.E.M. The absolute total distance (in meters) covered by individuals during the 3-min time slots was compared among trials using a linear mixed model (LMM) framework. To gain statistical robustness, we handled the five control-*vs*-treated trials (cypermethrin, fipronil, tau-fluvalinate, tetramethrin, thiamethoxam) simultaneously as a part of the same model, followed by post-hoc pairwise comparisons with Bonferroni *p*-value adjustments for multiple testing. In a preliminary step, we assessed the constancy and stability of the experimental design by comparing monitored distances among the five control groups only (simple linear model LM and Tukey multiple pairwise comparisons). In a second step, we introduced into the model the five treated groups and set the correct matching with their respective control group by specifying the trial identity as a random grouping factor (LMM and Dunnett multiple comparisons with control). We verified that the LMM normality and homoscedasticity assumptions were met by graphically inspecting model residuals and QQ-plots [30]. We further statistically confirmed residual normality (Shapiro-Wilk test, w = 0.98, p = 0.15) and variance homogeneity among all trials (Bartlett test, K² = 1.84, df = 4, p = 0.76) and all treatments (K² = 11.17, df = 9, p = 0.26). Statistical analyses were performed with the R software for statistical computing [31]. Fisher exact tests were performed with the JMP software (SAS) to compare mortality rates, assuming significant differences for P<0.01.

## Results

### Determination of sublethal doses

Sublethal doses (SLD_48h_) were determined from mortality assays preceding the locomotion tests. Two criteria were mandatory in our experiments to select experimental SLD_48h_: i) a dose producing a mortality level not statistically different from the control was considered as a SLD_48h_ and ii) twice the chosen dose (SLD_48h_) had to produce a mortality level significantly higher than the control. SLD_48h_ for each insecticide are given in S1 Table, with results of the statistical analysis on mortality assays (p-values from exact Fisher tests). SLD_48h_ were 2.5, 33 and 70 ng for the three pyrethroids cypermethrin, tau-fluvalinate and tetramethrin respectively. SLD_48h_ were 3.8 and 0.5 ng for thiamethoxam and fipronil respectively. Mortality levels after insecticide exposure were not corrected for control mortality levels [26], which were low in all series (0–2.5%).

### Locomotion in control bees

**Fig 2:**
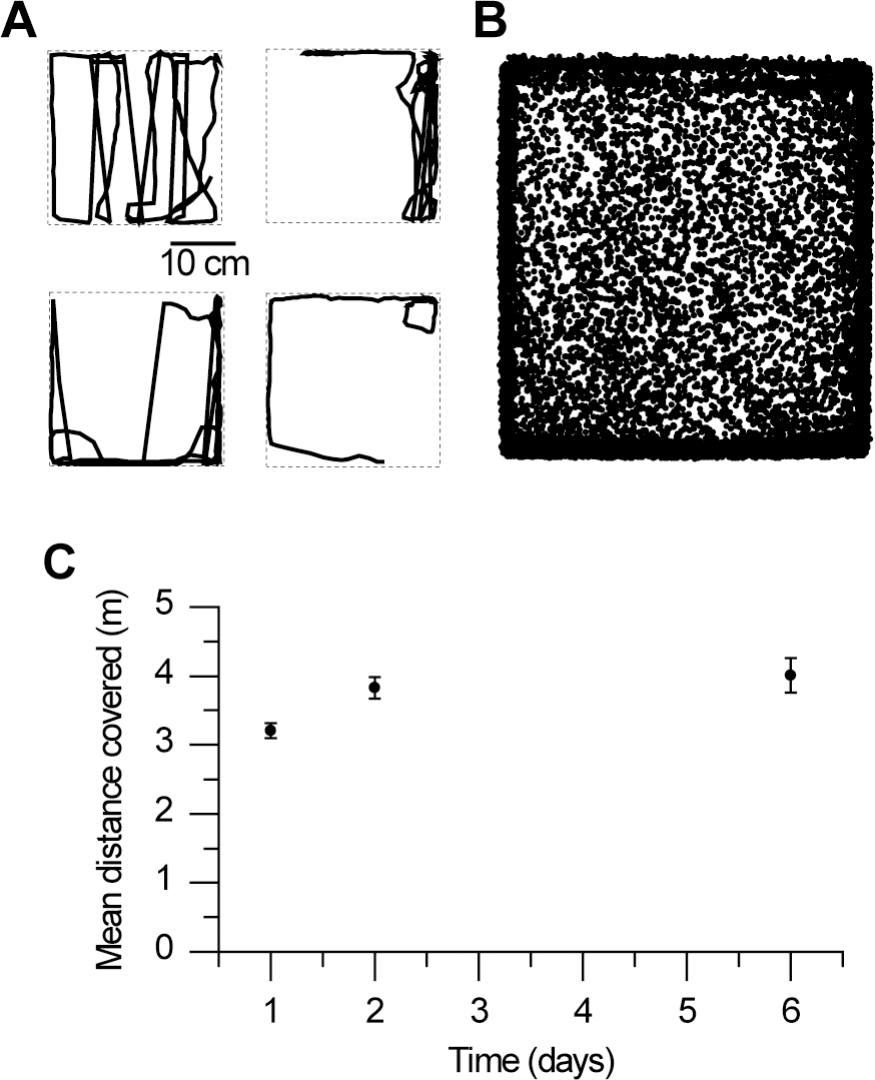
Video tracking of bees using a vertical arena. **A,** Examples of paths followed during 3 minutes by four individual young bees (day 1 after emergence). **B,** Superimposed paths followed by eighty individual bees. Overall, arena sides were more frequently visited. **C,** Locomotor ability measured at day 1, 2 and 6 after emergence (bees kept in an incubator). Mean distance (± S.E.M) covered by bees slightly increased from 3.2 to 3.8 meters between day 1 and 2 (p<0.01, n = 138 and 63 respectively) and did not significantly further increase as shown at 6 days after emergence (n = 38).

Locomotor function and deficits produced after exposure to an insecticide were evaluated by video tracking bees placed in a closed vertical arena. Individual honeybees subjected to this assay displayed variable trajectories, as illustrated for four individuals (first day after emergence) monitored for 3 minutes at a frequency of 1 Hz (Fig 2A). During 3 minutes, each bee can explore only a fraction of the arena. However, overall, bees visited all parts of the arena, although sides were visited more often, possibly owing to a positive thigmotaxis phenomenon (Fig 2B, superimposed trajectories of 80 control bees). The total distance covered was chosen as a proxy for the bee’s locomotor ability. In laboratory conditions, a bee’s locomotor ability only slightly increased with age as shown by distances measured from bees kept in a cage for 6 days (Fig 2C). Mean distance covered by bees increased from 3.2 ± 1.0 to 3.8 ± 1.6 m between day 1 and 2 (Mann-Whitney U = 3119, n_1_ = 138, n_2_ = 63, p<0.01) and did not significantly further increase at day 6 after emergence (4.0 ± 2.5 m, n = 38). Bees already showed good locomotion skills at day one after emergence, making this age suitable for the following locomotion assays on insecticide-treated bees.

### Locomotion in bees exposed to a SLD_48h_

**Fig 3:**
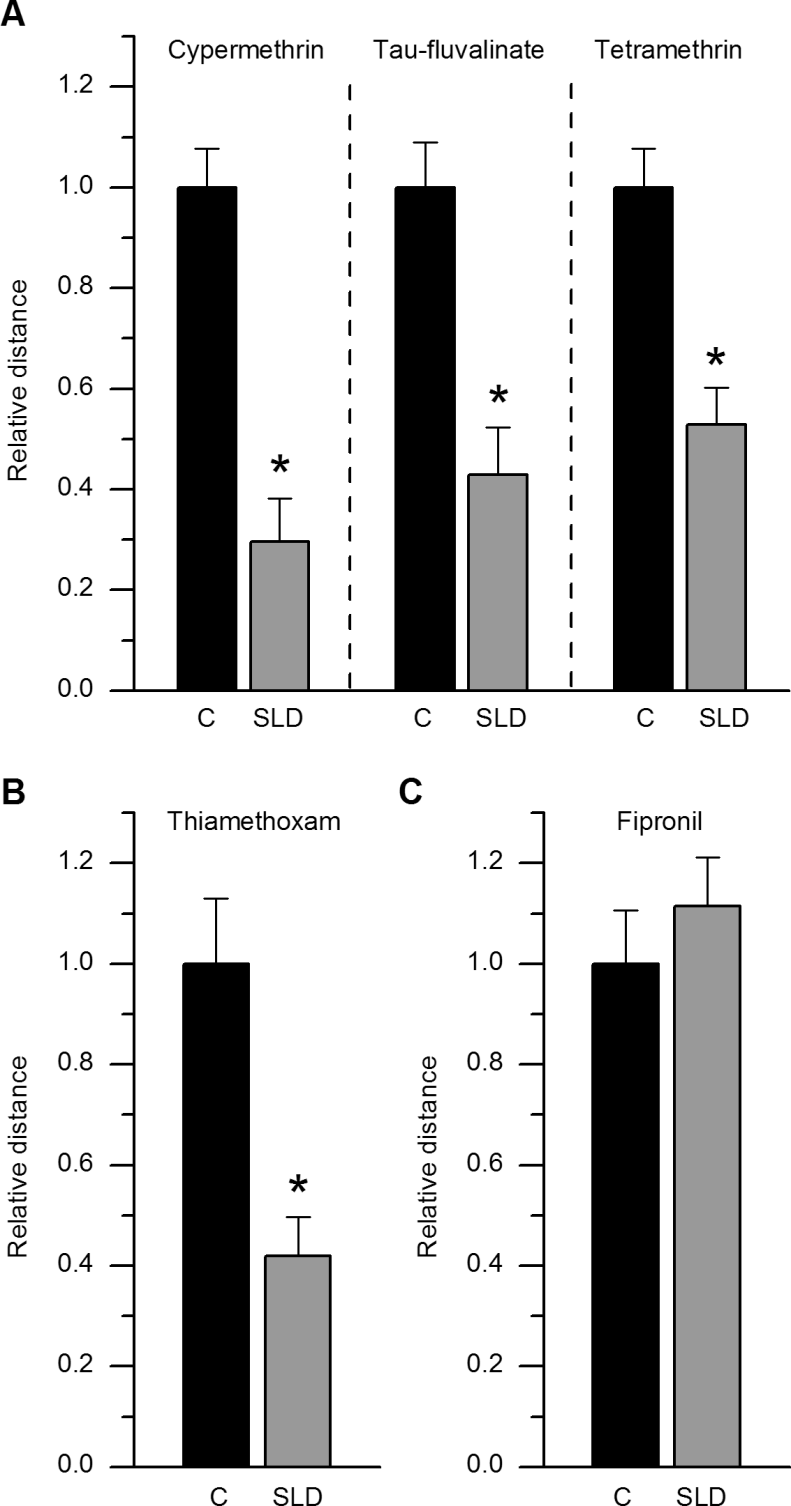
Evidence for locomotor deficits after exposure to a sublethal dose (SLD_48h_) of a pyrethroid or a neonicotinoid but not a phenylpyrazole. **A**, The average (± S.E.M) relative distance covered by young bees is significantly decreased 6±2h after exposure to either a SLD_48h_ of cypermethrin (2.5 ng/bee), tau-fluvalinate (33 ng/bee) or tetramethrin (70 ng/bee). **B**, A significant decrease in distance is observed after exposure to a SLD_48h_ of thiamethoxam (3.8 ng/bee) as well. **C**, The relative distance covered by bees after exposure to a SLD_48h_ of fipronil (0.5 ng/bee) is similar to the distance covered by control bees. In the case of fipronil, whereas early deleterious effects cannot be evidenced by the locomotion assay, an increased mortality is observed five days after exposure. For cypermethrin, n = 19 control and n = 20 exposed bees respectively. For tau-fluvalinate, n = 12 control and n = 19 exposed bees respectively. For tetramethrin, n = 20 control and n = 20 exposed bees respectively. For thiamethoxam, n = 19 control and n = 19 exposed bees respectively. For fipronil, n = 19 control and n = 20 exposed bees respectively.

Average distances covered by young bees (day 1 after emergence) were measured after exposure to an SLD_48h_ of one of the three pyrethroids: cypermethrin (2.5 ng/bee), tau-fluvalinate (33 ng/bee) and tetramethrin (70 ng/bee). For ease of comparison, distances covered by exposed bees were standardized to the average distance covered by corresponding control bees, set at 1 (Fig 3, relative control distances in black). S1 Fig also reports individual actual distances in meters (S1 Fig). All the five control groups delivered statistically identical locomotion properties, with no significant distance variation in any pairwise combination of trial (S2 Table, S2 Fig). Given the experimental stability of control groups, we could readily assess the five treatments as a part of a single LMM model. The distance covered by individuals was significantly lower in all treated groups compared to control, except for the fipronil trial (S2 Table, S2 Fig). In bees exposed to an SLD_48h_ of cypermethrin, the mean covered distance was significantly decreased by 71 ± 9% relative to control bees (Fig 3, S1 Fig, S2 Table). Tau-fluvalinate was also very potent at the SLD_48h_, and the covered distance was significantly diminished by 58 ± 10% (Fig 3, S1 Fig, S2 Table), while tetramethrin (70 ng/bee) significantly decreased the distance covered by 48 ± 7% (Fig 3, S1 Fig, S2 Table). At the chosen SLD_48h_, all 3 pyrethroids appear qualitatively equally potent. Exposure to an SLD_48h_ of the neonicotinoid thiamethoxam (3.8 ng/bee) resulted in a similar significant decrease by 58 ± 8% of the distance covered (Fig 3, S1 Fig, S2 Table). Exposure to an SLD_48h_ of fipronil (0.5 ng/bee) did not produce any significant effect on locomotion (Fig 3, S1 Fig, S2 Table).

### Mortality several days after exposure to fipronil in the absence of early locomotor deficits

In parallel with locomotion assays, long-term survival was measured after exposure to the insecticide SLD_48h_, to explore whether the locomotor deficits that we have measured could induce any mortality several days after exposure in laboratory conditions. The current legislation imposes that acute contact mortality tests have to be routinely performed for 48h [26]. However, if mortality increases by more than 10% between 24 and 48 h, the assay should be extended up to 96 h. Here, for all pyrethroids and the neonicotinoid tested, mortality rates were stable between 24 and 48 h. We found that the SLD_48h_ was sublethal at 120 h as well, indicating that the early locomotor deficit observed does not compromise survival five days after exposure, at least in a controlled laboratory environment. At 120 h, the SLD_48h_ of cypermethrin, tetramethrin, tau-fluvalinate or thiamethoxam did not induce mortality more than their respective controls (Fisher exact tests, P>0.14). Interestingly, whereas fipronil was the only modality in which no locomotor deficits were detected, with a mortality rate stable between 24 and 48 h (1 and 2% respectively, P = 0.6515), the SLD_48h_ of this insecticide started to produce an increased mortality at 72 h (14%, P<0.0001 as compared to 48 h) and a high mortality rate at 120 h after exposure (78%, n = 180 bees) as compared to control bees (1.5%, n = 180 bees, P<0.0001). It is noteworthy to mention that the survival of control bees was stable between 48h and 120h (99.5% and 98.5% respectively, n = 180, P = 0.6229).

## Discussion

For pollinators, sublethal effects of insecticides increase toxicological risks and thus should be taken more into account in the methods of risk evaluation [32]. For the first time, we analyzed the sublethal effects of pesticides from three major insecticide classes on young bees (day 1 after emergence) in a standardized honeybee walking locomotion test. Emphasis has been put on pyrethroids that were much less studied than the two other insecticide classes despite 1) their high toxicity towards insects, 2) their pervasive use in agriculture and 3) their prevalence in hives. Specific experimentally-determined sublethal doses were selected for each insecticide (see Methods). In our study, assuming a mean individual bee weight of 0.1 g [1], the SLD_48h_ of pyrethroids were 25 ppb for cypermethrin, 330 ppb for tau-fluvalinate and 700 ppb for tetramethrin. By comparison, quantitatively similar values of 13 pyrethroid residues have been detected in North American hives [1]. For instance, in foundation wax, cypermethrin and fluvalinate were found in 23.8 and 100% of samples at maximal levels of 131 and 10120 ppb respectively (average levels 51.6 and 2006 ppb respectively). Knowing that multiple pyrethroids can be found in the same hive, preimaginal bees and newly emerged bees were thus potentially exposed to cumulated doses [1] that are compatible with SLD_48h_ used in the present study. However, the gaps in the current knowledge on pesticides toxicokinetics (e.g. on the transfer rate of pesticides from hive matrices to the body of a young bee) precludes comparing the level of exposure resulting from contact with contaminated waxes and the level of exposure resulting from a laboratory procedure in which a droplet of contaminated solution is applied on the thorax. Currently, a model that would link these two modes of exposure is unfortunately lacking for a risk evaluation to be accurately performed. For fipronil, our SLD_48h_ was 5 ppb. By comparison, 1.4% of wax samples contained a maximum of 35.9 ppb of fipronil, 0.3% of pollen samples contained a maximum of 28.5 ppb [1]. For thiamethoxam, our SLD_48h_ was 38 ppb. By comparison, 0.3% of pollen samples contained a maximum of 53.3 ppb of thiamethoxam and it was not detected in wax [1].

An SLD_48h_ of all the insecticides tested (except fipronil) triggered serious locomotor deficits. According to available LD_50 at 48h_ values for cypermethrin, that vary from 25 to 121 ng/bee, [33, 34], an SLD_48h_ of cypermethrin 10 to 48 fold lower than the LD_50_ seriously impairs locomotion. The SLD_48h_ for tau-fluvalinate is between 75 and 600 fold lower than published LD_50_ values (2.5 μg—20 μg/bee, [34, 35]). In the case of tetramethrin, the effective SLD_48h_ was only two times lower than the available LD_50_ [36]. Very different maximal ratios between LD_50_ and SLD_48h_ values for the three tested pyrethroids (2, 48 and 600 for tetramethrin, cypermethrin and tau-fluvalinate, respectively) suggest that within a chemical family, deleterious effects of individual insecticides have to be evaluated separately. A similar locomotor deficit was observed with a SLD_48h_ of tau-fluvalinate and a 13 fold lower SLD_48h_ of cypermethrin. This result is consistent with a 20 fold lower LD_50_ obtained with cypermethrin than with tau-fluvalinate [34]. Tau-fluvalinate is commonly used against the bee parasite *Varroa destructor*. The locomotor deficits observed here after exposure to low doses of tau-fluvalinate surely challenge the widespread concept that it can safely be used in hives.

Since honeybees’ skeletal muscles do not express functional voltage-gated Na^+^ channels, locomotion deficits cannot be explained by a direct action of pyrethroids on such channels in muscle [37, 38]. Locomotor deficits can then be potentially attributed to non-mutually exclusive explanations. First, the locomotor deficits observed in our study could be a consequence of pyrethroid effects on sodium channels located in the central nervous system (in brain and other ganglia) impairing information processing and motor command [39]. Alternatively, impaired electrical activity of sensory neurons housed in the antennae, that are more pyrethroid-sensitive than central neurons, may affect sensory perception and thus impede locomotion [39, 40]. Structural differences between tau-fluvalinate, cypermethrin and tetramethrin in the acidic and alcohol moieties (Fig 1) may produce the different sets of interactions within the channel pore revealed by molecular modeling [41–43], thus giving molecular support for drug-specific modifications in the honeybee sodium channel kinetic parameters [39]. The cloning and expression of the honeybee voltage-dependent sodium channel, AmNa_v_1 [43] together with the analysis of the changes induced by the different pyrethroids using numerical simulation bring a set of important tools that will be useful to fully characterize and understand the binding differences between these pyrethroids and hence their differential toxicity. Deficits could also be related to pyrethroid potency on secondary targets such as voltage-gated calcium channels [44, 45] that are broadly distributed in honeybee tissues [46, 47]. Calcium channels underlie action potentials in muscles of the honeybee or other insects, [37, 38, 48, 49]. Effects on muscle calcium channels would thus not only impair locomotion but thermoregulation or hemolymph circulation as well. A direct effect on bee muscle cells has actually been shown *in vitro* [37]. Our recent cloning and expression of *Apis mellifera* calcium channels (AmCa_v_) will allow for more systematic testing of pyrethroids [46, 47].

To our knowledge, the identification of a strong walking deficit in young bees after contact exposure to an SLD_48h_ of thiamethoxam has never been reported before. At 1 ng/bee, no walking deficit was detected [22], whereas the SLD_48h_ used in the present study (3.8 ng/bee) clearly impairs locomotion. At an oral SLD of 1.3 ng/bee (i.e. 25–33 fold lower than the LD_50_, [34, 50]), a fraction of bees also fail to perform their normal homing flight [7]. The neonicotinoids primary mode of action has been studied in honeybee neurons and is compatible with neurotoxic actions on the nervous cholinergic system [51–54]. Several nicotinic receptor subtypes are involved in complex behaviors and memory processes, and may be differentially altered by sublethal doses of neonicotinoids [55]. Fipronil did not affect locomotion at the SLD_48h_ (see also [10]), but surprisingly induced significant mortality at 5 days after exposure. We did not observe this phenomenon with other insecticides (see also [34] for cypermethrin and thiamethoxam), strongly suggesting that it is insecticide-specific. Whereas SLD_48h_ of all three pyrethroids and the neonicotinoid clearly impair the distance covered by bees, analysis of more subtle behaviors could resolve undetected fipronil-induced deficits. For instance, longer recording durations could reveal subtle alterations in inter-individual interactions, grooming behaviors and time spent near a food source [25, 29, 56].

In conclusion, the locomotion test allowed the identification of important deficits in young bees. It revealed that these effects are insecticide-specific and cannot be simply extrapolated from LD_50_ values. This assay could thus be used as a preliminary analysis before implementing more sophisticated homing-flight experiments or more subtle memory or orientation tests [7, 18, 57]. It is worth noting that such a laboratory locomotion test is formalized, standardized and displays the least sensitivity to seasonal, phenologic, weather and landscape variations [58]. The recent temporary ban of neonicotinoids in Europe, due to their high toxicity towards the honeybee, calls for alternative methods of pest control, which thus become a priority for modern agriculture, but also a societal issue. Pyrethroids, that already represent one fifth of the global pesticides market [59], have already been used as an alternative solution to restricted or banned pesticides. Their toxicity identified using a simple locomotion test suggests that pyrethroids can be as toxic as a neonicotinoid towards bees, and therefore implies that the molecules to be used would need to be carefully selected.

## Supporting Information

S1 Fig Individual distances covered by bees in each group. Individual distances (in meters) covered by control bees and exposed bees are plotted as white and grey dots respectively, for each insecticide. Average distances (± S.E.M) are shown for each modality. Mean distances in control groups were similar (3.14 ± 0.24 m, 3.26 ± 0.29 m, 3.50 ± 0.27 m, 3.22 ± 0.42 m, 3.37 ± 0.35 m for cypermethrin, tau-fluvalinate, tetramethrin, thiamethoxam and fipronil respectively, see S2 Table for statistics). Mean distances after exposure to a SLD_48h_ were 0.93 ± 0.27 m, 1.40 ± 0.31 m, 1.85 ± 0.26 m, 1.35 ± 0.25 m, 3.22 ± 0.33 m for cypermethrin, tau-fluvalinate, tetramethrin, thiamethoxam and fipronil respectively (see Fig 3 for numbers of bees in each group).
(TIF)

S2 Fig Effect size estimates for variations of distance covered by individuals (a) among control groups of the five trials and (b) between treatment and control groups. Horizontal bars stand for the 95% confidence intervals returned by the post-hoc multiple pairwise comparisons. The vertical dashed line indicates the no-effect level.
(TIF)

S3 Fig Observed average instantaneous speed during the 3-min recording time as compared to the 95% confidence limits (shaded area) for an expected steady-state average speed. The instantaneous speed during the 3-min recording time was measured in a pilot study performed on 80 non-exposed individual bees. The mean instantaneous speed (mm.s-1) was averaged per 10-s slots among the 80 individual bees. We compared the observed averages with the 95% confidence interval (CI) range expected under the hypothesis of steady-state average instantaneous speed. The 95% CI range was obtained from a bootstrapping procedure whereby the speed data were randomly shuffled along the temporal axis. We recomputed 200 random rearrangements of the raw database and then extracted the average speed values at the 2.5% and 97.5% ranks for each 10s step to delineate the 95% CI. Average speed tended to decrease as time lapses, with observed values being closer to (or slightly above) the upper CI boundary during the first minute of recording, and closer to the lower CI boundary during the third minute of recording. At the very last 10s recording slot, average speed fell below the expected steady-state confidence limits. We therefore considered that the 3-min standard recording duration was appropriate to cover statistically steady-state locomotion samples in our control-*vs*.-treated experiments.
(TIF)

S1 Table Mortality tests for the determination of sublethal doses. (XLSX)

S2 Table Statistical outputs of LM and LMM models comparing distances covered by individuals (m) among control groups of the five trials, and between treatments. The post-hoc pairwise comparisons indicate that only the fipronil treatment did not significantly affect distances. See S2 Fig for effect size estimates.
(DOCX)
