# Supplementary figures and images for: A Locomotor Deficit Induced by Sublethal Doses of Pyrethroid and Neonicotinoid Insecticides in the Honeybee Apis mellifera
Source: PLoS One. 2015 Dec 14;10(12):e0144879. doi: 10.1371/journal.pone.0144879 (PMC4682844; doi:10.1371/journal.pone.0144879)

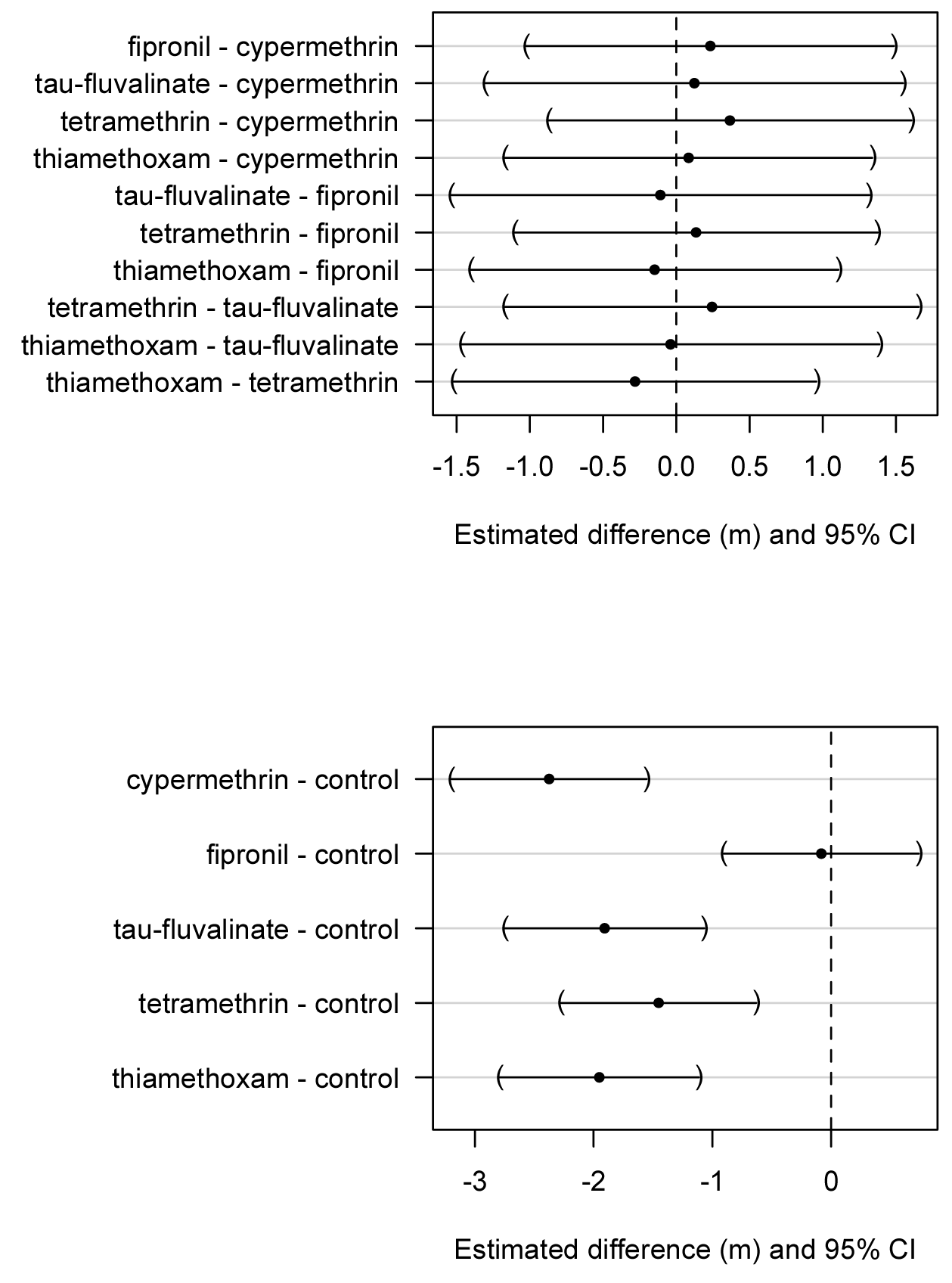

Supplement: S2 Fig — Horizontal bars stand for the 95% confidence intervals returned by the post-hoc multiple pairwise comparisons. The vertical dashed line indicates the no-effect level. (TIF) [file pone.0144879.s002.tif]

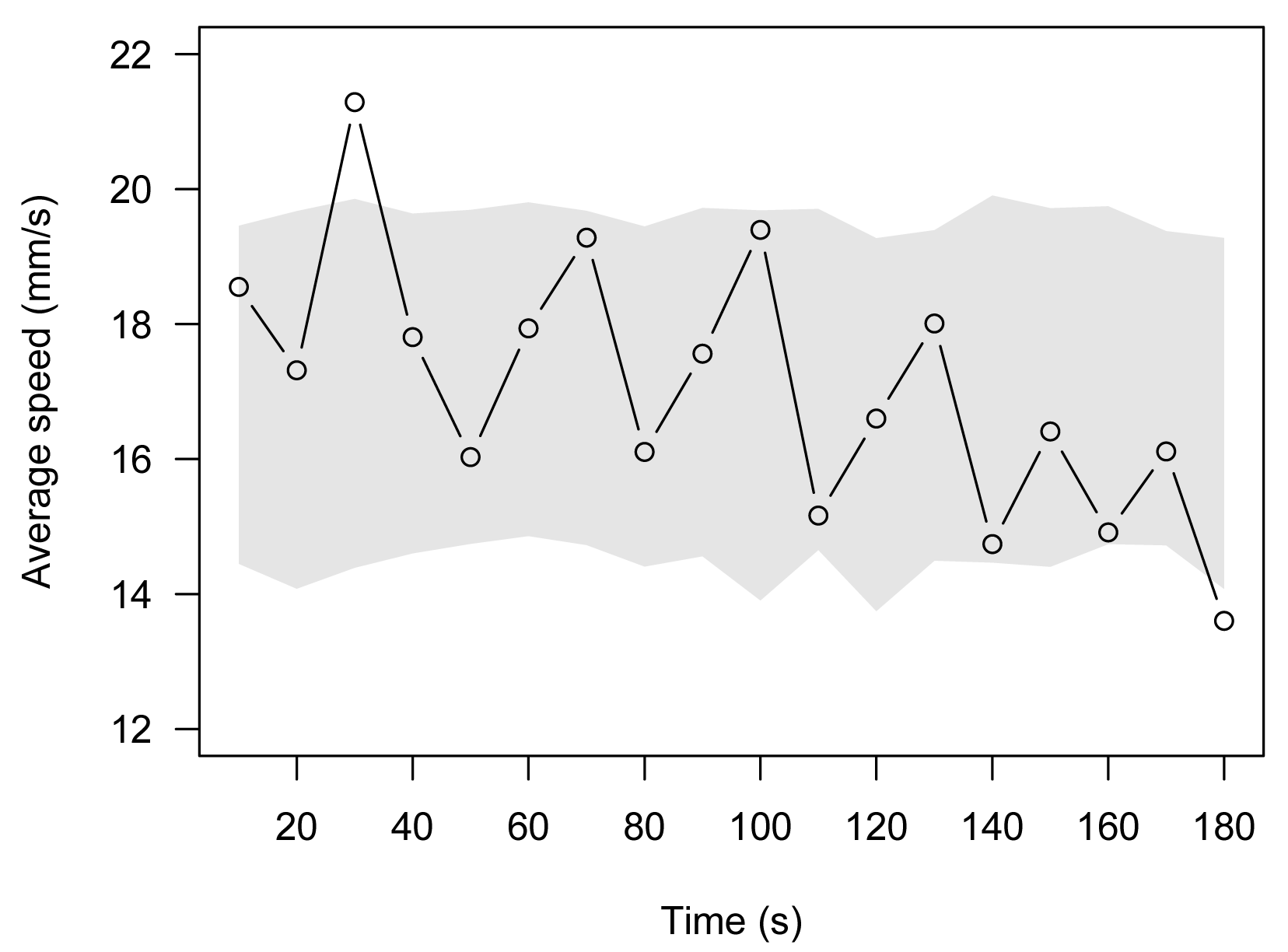

Supplement: S3 Fig — The instantaneous speed during the 3-min recording time was measured in a pilot study performed on 80 non-exposed individual bees. The mean instantaneous speed (mm.s-1) was averaged per 10-s slots among the 80 individual bees. We compared the observed averages with the 95% confidence interval (CI) range expected under the hypothesis of steady-state average instantaneous speed. The 95% CI range was obtained from a bootstrapping procedure whereby the speed data were randomly shuffled along the temporal axis. We recomputed 200 random rearrangements of the raw database and then extracted the average speed values at the 2.5% and 97.5% ranks for each 10s step to delineate the 95% CI. Average speed tended to decrease as time lapses, with observed values being closer to (or slightly above) the upper CI boundary during the first minute of recording, and closer to the lower CI boundary during the third minute of recording. At the very last 10s recording slot, average speed fell below the expected steady-state confidence limits. We therefore considered that the 3-min standard recording duration was appropriate to cover statistically steady-state locomotion samples in our control-vs.-treated experiments. (TIF) [file pone.0144879.s003.tif]
