# Supplementary material for: A Locomotor Deficit Induced by Sublethal Doses of Pyrethroid and Neonicotinoid Insecticides in the Honeybee Apis mellifera
Source: PLoS One. 2015 Dec 14;10(12):e0144879. doi: 10.1371/journal.pone.0144879 (PMC4682844; doi:10.1371/journal.pone.0144879)
Supplement: S2 Table — The post-hoc pairwise comparisons indicate that only the fipronil treatment did not significantly affect distances. See S2 Fig for effect size estimates. (DOCX) [file pone.0144879.s005.docx]

| Model | Statistic | *P*-value |
| --- | --- | --- |
| Comparing control groups among trials (LM) | F_4,84_ = 0.202 | 0.936 |
| Assessing treatment effects (LMM) | F_5,177_ = 20.11 | <0.001 |
| Post-hoc Dunnett pairwise comparisons with control:  cypermethrin *vs.* control  fipronil *vs.* control  tau-fluvalinate *vs.* control  tetramethrin *vs.* control  thiamethoxam *vs.* control | z = -7.42  z = -0.26  z = -5.83  z = -4.54  z = -5.97 | <0.001  0.999  <0.001  <0.001  <0.001 |
